# Vaccine effectiveness of cell-culture relative to egg-based inactivated influenza vaccine during the 2017-18 influenza season

**DOI:** 10.1371/journal.pone.0229279

**Published:** 2020-02-26

**Authors:** Nicola P. Klein, Bruce Fireman, Kristin Goddard, Ousseny Zerbo, Jason Asher, James Zhou, James King, Ned Lewis

**Affiliations:** 1 Kaiser Permanente Vaccine Study Center, Kaiser Permanente Northern California Division of Research, Oakland, California, United States of America; 2 U.S. Biomedical Advanced Research and Development Authority (BARDA), Office of the Assistant Secretary for Preparedness and Response, United States Department of Health and Human Services, Washington, District of Columbia, United States of America; University of South Dakota, UNITED STATES

## Abstract

There is concern that influenza vaccine effectiveness (VE) may be attenuated by passage in eggs during manufacture. We compared quadrivalent cell-culture vaccine with egg-based vaccines, most of which were trivalent, against influenza A and B during 2017–2018 when A(H3N2) and B/Yamagata (present only in quadrivalent vaccines) predominated. We retrospectively examined risk of PCR-confirmed influenza A and B in members of Kaiser Permanente Northern California aged 4–64 years. We estimated the relative VE (rVE) of cell-culture vaccine versus egg-based vaccines, and the absolute VE (aVE) of each vaccine comparing vaccinated to unvaccinated individuals. Analyses used Cox regression with a calendar timeline, stratified by birth year, and adjusted for demographics, co-morbidities and utilization. One-third (1,016,965/3,053,248) of the population was vaccinated; 932,545 (91.7% of vaccinees) received egg-based and 84,420 (8.3%) received cell-culture vaccines. The rVE against influenza A was 8.0% (95% CI: –10, 23); aVE was 31.7% (CI: 18.7, 42.6) for cell-culture and 20.1% (CI: 14.5, 25.4) for egg-based vaccines. The rVE against influenza B was 39.6% (CI: 27.9, 49.3); aVE was 40.9% (CI: 30, 50.1) for cell-culture and 9.7% (CI 3.5, 15.6) for egg-based trivalent vaccines. Inclusion of the B/Yamagata lineage in the quadrivalent cell-based vaccine provided better protection against influenza B but vaccine effectiveness against influenza A was low for both the cell-culture vaccine and the egg-based vaccines. Improving influenza vaccines requires ongoing comparative vaccine effectiveness monitoring.

## Introduction

In June 2018, the Centers for Disease Control and Prevention (CDC) reported that influenza vaccine effectiveness (VE) against A(H3N2) influenza virus for the 2017–2018 season was ~22% [1], lower than the approximately 40% (and under) range observed in recent years [2–7].

One hypothesis for the low VE observed during the 2017–2018 influenza season was that it may have been related to genetic changes which could have occurred on the hemagglutinin protein component of the influenza A(H3N2) vaccine virus when it was passaged in eggs to make the influenza A vaccine component [8, 9]. Such genetic changes could potentially result in an influenza vaccine that is less effective against circulating influenza A(H3N2) viruses [10–12].

Flucelvax^™^ (Seqirus), a cell-culture inactivated influenza vaccine (ccIIV), is not manufactured in eggs. Trivalent cell-culture vaccine was initially licensed in the U.S. in November 2012 for people aged 18 and older [13]. In May of 2016 the FDA approved a quadrivalent formulation (ccIIV4) for people 4 years and older [14]. In August 2016, FDA approved cell-culture candidate influenza vaccine viruses for use in manufacturing influenza vaccines [15]. Before this time, influenza seed viruses, even for ccIIV, were adapted for growth in eggs prior to vaccine manufacturing. However, the influenza A(H3N2) vaccine virus component for the 2017–2018 cell-culture vaccine was propagated solely in cell culture, though the A(H1N1) and B components were still propagated in eggs [16].

Since ccIIV4 avoided any egg-adapted changes in the influenza A(H3N2) component, the question of whether the ccIIV4 was more effective during the 2017–18 season than were standard dose egg-based influenza vaccines (SD-IIV) has been raised. During the 2017–2018 influenza season, in California approximately 84% of the circulating influenza was A(H3N2) [17, 18] while B Yamagata lineage was the predominant circulating type B virus [19].

The aims of our study were to investigate within Kaiser Permanente in Northern California (KPNC) during the 2017–2018 influenza season whether the VE of ccIIV4 against influenza differed from that of SD-IIV. We estimated the relative VE (rVE) of ccIIV4 versus trivalent or quadrivalent SD-IIV (SD-IIV3/4) and the absolute VE (aVE) of ccIIV4 and SD-IIV3/4 against all confirmed influenza A. We also assessed the rVE of ccIIV4 versus SD-IIV3 and the aVE of ccIIV4 and SD-IIV3 against influenza B. For this aim, because SD-IIV3 only included influenza B Victoria lineage while ccIIV4 included both influenza B Victoria and Yamagata lineages. we hypothesized that ccIIV4 would be more effective against the B Yamagata lineage than SD-IIV3.

## Methods

### Study setting

KPNC is an integrated health care system with a membership of approximately 4 million in 2018, including approximately 3 million members between 4 to 64 years of age. Members receive nearly all their care at KPNC facilities, which includes 46 medical clinics and 21 hospitals. All healthcare utilization, diagnoses, laboratory tests, vaccines, and medications are captured in KPNC’s electronic medical record. Vaccination data within the electronic medical record includes the date of vaccination, brand, lot, dose, and injection site. KPNC members comprise more than 30% of the population and are representative of the underlying racial, ethnic, and socioeconomic population of Northern California, although they somewhat underrepresent those at the very lowest incomes.

### Study population

This study included all individuals who were KPNC members during 2017–2018 influenza season and who were aged 4–64 years. We limited the study population to those aged > 4 because ccIIV4 is only licensed for those >4 years of age and to those aged <65 years because KPNC targeted use of high-dose IIV3 among those >65 years of age.

The study was approved by the KPNC Institutional Review Board with a waiver of written informed consent because the study had no direct contact with study participants.

### Influenza infection

We defined an influenza infection as a positive combined polymerase chain reaction (PCR) test result for influenza A or B (GeneXpert PCR assay). All PCR results are captured electronically within the medical record and we included all individuals who tested positive for influenza. The decision regarding whether to PCR-test for influenza was at the discretion of each clinician. PCR testing was limited to detection of influenza A and B; no A subtyping or B genotyping was performed.

### Statistical analysis

We conducted rVE analyses using two different epidemiologic study design frameworks, both of which included only individuals who received influenza vaccine. The primary analysis was a vaccinated cohort design which included all KPNC vaccinees aged 4–64 years. We estimated the risk of PCR-confirmed influenza among members receiving ccIIV4 versus those receiving SD-IIV by comparing the proportion ccIIV4 among all vaccinees in those with a positive PCR test with the proportion ccIIV4 among all vaccinees regardless of testing. The supplemental second analysis used a test-negative design which included only vaccinated members aged 4–64 years who had undergone PCR testing for influenza [20–22]. In this supplemental analysis, we estimated the risk of test-positive influenza by comparing the odds of ccIIV4 vaccination among the same PCR-confirmed influenza cases with the odds of ccIIV4 vaccination among PCR-negative KPNC vaccinees.

We used Cox regression models for the cohort analyses. Potential covariates were evaluated for inclusion in the models by examining the bivariate associations with type of vaccine (for rVE) or being vaccinated (for aVE) and with being tested for influenza. In addition, for each model we examined the influence on VE estimates of each variable included singly with vaccine exposure in a model stratified by birth year (only history of inpatient admissions changed VE parameter estimates by more than 5% on log[HR] scale). The other covariates were included on the basis of being related to both exposure and outcome or due to pre-specified plausible association.

The models were specified with a calendar timeline, stratified by birth year (which closely adjusts for age), and with covariate adjustment for KPNC facility, sex, race, years of KPNC membership, prior season influenza vaccine, number of inpatient admissions in prior year, the number of weeks with outpatient visits in the prior year, and co-morbidities in the prior 2 years (asthma, diabetes, chronic obstructive pulmonary disease [COPD], and coronary heart disease). We adjusted for these comorbidities because they were associated with higher risk of influenza disease, being tested for influenza or with receiving an influenza vaccine. The model used a calendar timeline to account for the exact calendar date of each influenza case. This was important because the intensity of the influenza epidemic varied over the course of the season; it also accounted for changes in the proportion of ccIIV vaccinees over time. Follow-up began 7 days after vaccination and continued until the end of influenza season, a positive PCR test for influenza, disenrollment from KPNC, or death, whichever occurred first. The Cox models estimated the hazard of influenza and VE estimates were calculated as 1 minus the hazard ratio (HR).

In the test-negative analyses, we compared the odds of vaccination in PCR-confirmed cases versus the odds of vaccination in individuals who were test-negative among vaccinees only. For all the test negative analyses, we used logistic regression and adjusted the results for that same covariates as in the Cox regression analysis including calendar time (in months, and half-months in December and February if feasible) and age (in decades). We calculated VE as 1 minus the adjusted odds ratio (OR).

We compared ccIIV4 with SD-IIV3/4 to estimate the rVE against influenza A and ccIIV4 with SD-IIV3 to estimate the rVE against influenza B.

To estimate aVE, we used the same cohort and test-negative design frameworks but also included unvaccinated individuals in the analyses. For aVE analyses which compared vaccinated with unvaccinated, individuals contributed unvaccinated follow-up time until receipt of influenza vaccine or the end of their study enrollment period. We separately compared ccIIV4 and SD-IIV3/4 vaccinees with unvaccinated persons to estimate aVE against influenza A and compared ccIIV4 and SD-IIV3 vaccinees with unvaccinated persons to estimate aVE against influenza B.

### Power calculation

For the cohort rVE analysis, we estimated that we had 80% power to detect approximately 20% rVE by the end of the 2017–2018 influenza season, using a likelihood ratio test, 2-sided alpha = 0.05. For the test-negative rVE analysis, comparing the odds of ccIIV4 vaccination in the same PCR-confirmed breakthrough cases with test-negative vaccinees, we estimated that we had slightly more than 80% power to detect 22–23% rVE by the end of the 2017–2018 influenza season.

## Results

**Fig 1:**
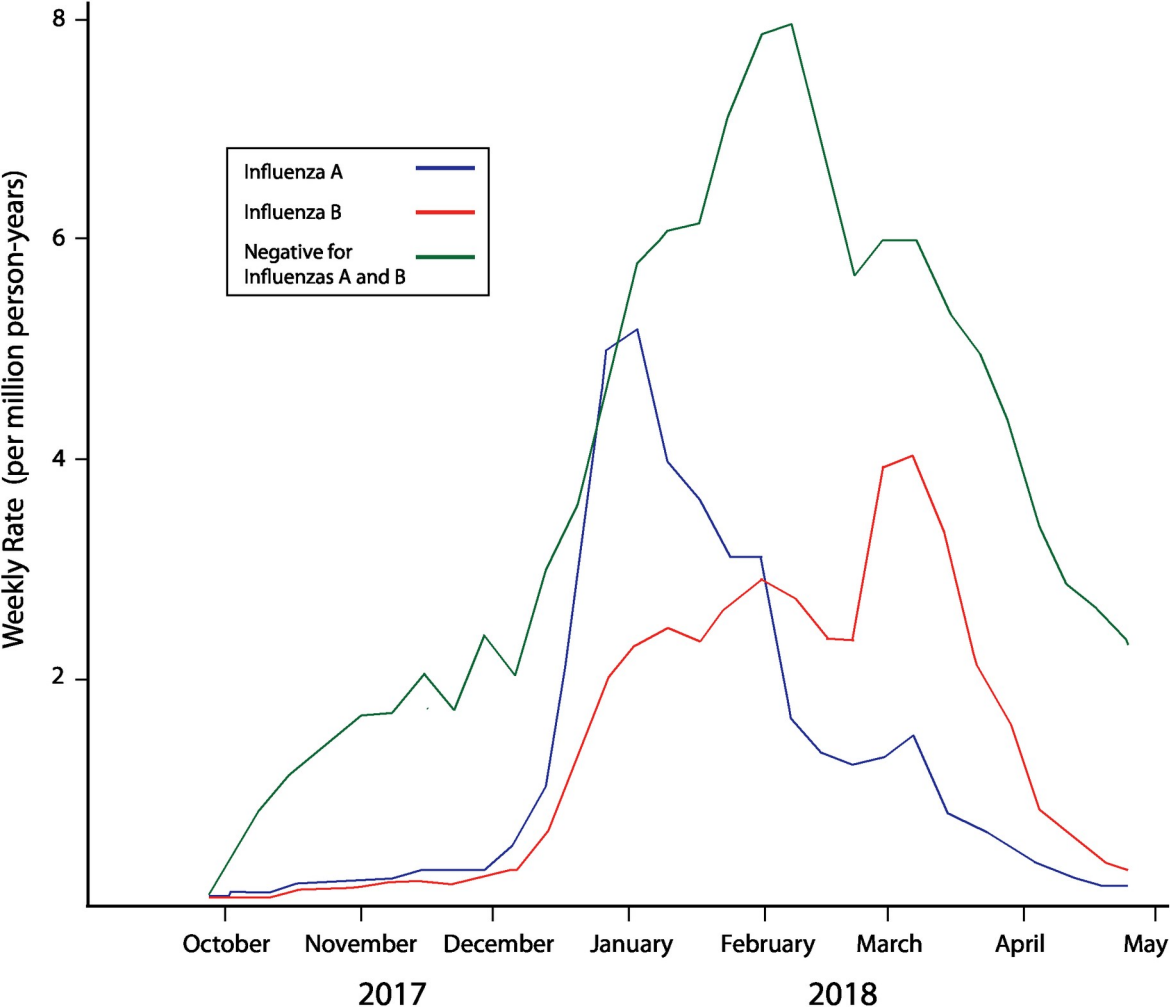
Rates of Influenza A and B in the Study Population by Week, Kaiser Permanente Northern California 2017–18. Blue line denotes weekly rate of influenza A (per million person-years per week), red line denotes weekly rate of influenza B (per million person-years per week), and green line denotes the weekly rate of negative influenza A and B tests (per million person years per week).

Both influenza A and B circulated within KPNC during the 2017–18 season, with influenza A activity peaking sharply in late December/early January and continuing through March/April 2018. In contrast, influenza B circulation, although it began in December, slowly increased until it peaked in early March, gradually declining through late April/May 2018 (Fig 1).

The study included 3,053,248 persons aged 4–64 years, of whom 2,036,283 (66.7%) were unvaccinated and 1,016,965 (33.3%) received an influenza vaccine. Among those vaccinated, 803,813 (79%) received SD-IIV3, 128,732 (12.7%) received SD-IIV4 (for a total of 932,545 in the SD-IIV3/4 group), and 84,420 received ccIIV4 (8.3%). Vaccine uptake rose steeply in the study population for both ccIIV4 and SD-IIV3/4 vaccines starting in October 2017 and continuing into February 2018. However, uptake of ccIIV4 and SD-IIV3/4 was not identical and the relative coverage of ccIIV4 versus SD-IIV3/4 vaccines varied somewhat over time (S1 Fig). Starting in December 2017, the percent of all influenza vaccines that was ccIIV4 increased from ~7% to ~8% in March 2018, which represents a 14% increase in the proportion of all IIV that was ccIIV4 (S2 Fig).

Demographic characteristics and comorbidities were generally similar between the ccIIV4 and SD-IIV3/4 populations (Table 1). However, a greater proportion of ccIIV4 vaccinees was 4–17 year olds (51.8% vs 23.6% of SD-IIV3/4 vaccinees and 18.7% of unvaccinated), resulting in modest imbalances in co-morbidities related to age differences. In addition, a higher proportion of vaccinated than unvaccinated individuals had received an influenza vaccine in the prior year (66.2% of SD-IIV3/4 and 60.4% of ccIIV4 vaccinees vs 13.5% of unvaccinated).

**Table 1:** Study population characteristics among 4-64-year-olds, Kaiser Permanente Northern California 2017–18.

	Received ccIIV4^a^ N = 84,420 (%)	Received SD-IIV3/4^b^ N = 932,545 (%)	Unvaccinated N = 2,036,283 (%)
Sex			
Female	48,063 (56.9)	525,772 (56.4)	973,855(47.8)
Male	36,357 (43.1)	406,773 (43.6)	1,062,428 (52.2)
Age (years)			
4 to 17	43,735 (51.8)	220,419 (23.6)	380,305 (18.7)
18 to 64	40,685 (48.2)	712,126 (76.4)	1,655,978 (81.3)
Race/ethnicity^c^			
White	33,287 (39.4)	416,797 (44.7)	875,579 (43.0)
Hispanic	23,068 (27.3)	199,104 (21.4)	492,978 (24.2)
Asian	22,069 (26.1)	260,533 (27.9)	371,592 (18.2)
Black	6,675 (7.9)	53,005 (5.7)	177,728 (8.7)
Pacific Islander/Hawaiian	1,008 (1.2)	8,960 (1.0)	21,097 (1.0)
Native American/Alaskan	800 (0.9)	8,809 (0.9)	18,113 (0.9)
Multiracial	5,059 (6.0)	47,337 (5.1)	83,084 (4.1)
Comorbidities			
Coronary Heart Disease	533 (0.6)	11,453 (1.2)	12,137 (0.6)
Chronic Obstructive Pulmonary Disease	310 (0.4)	6,528 (0.7)	6,073(0.3)
Asthma	12,175 (14.4)	120,248 (12.9)	174,131 (8.6)
Diabetes	3746 (4.4)	76554 (8.2)	87,236 (4.3)
Received influenza vaccine in prior year	50,962 (60.4)	617,441 (66.2)	275,510 (13.5)
Hospital admission in the prior year	3,594 (4.3)	39,506 (4.2)	54,509 (2.7)
KPNC membership >1 year	74,303 (88.0)	822,560 (88.2)	1,673,873 (82.2)

^a^ccIIV4 = quadrivalent cell-culture inactivated influenza vaccine.

^b^SD-IIV3/4 = trivalent or quadrivalent inactivated influenza vaccines

^c^ Percentages may total over 100 because individuals can report multiple races within KPNC.

Among the 84,420 who received ccIIV4, 83,023 contributed vaccinated follow-up time, 925 (1.11%) were tested and 141 (0.17%) tested influenza A positive. Among 932,545 who received SD-IIV3/4, 919,903 contributed vaccinated follow-up time, 10,007 (1.09%) were tested and 1571 (0.17%) tested influenza A positive. Among the 2,036,947 who contributed unvaccinated time, 17,473 (0.86%) were tested and 4088 (0.20%) tested influenza A positive. Comparing ccIIV4 with SD-IIV3/4, the adjusted HR for risk of influenza A was 0.92 (95% CI 0.77, 1.10), which corresponds to a rVE of 8% (95% CI -10, 23; Table 2). Compared with unvaccinated, the adjusted aVE against influenza A was 31.7% (95% CI 18.7, 42.6) for ccIIV4 and 20.1% (95% CI 14.5, 25.4) for SD-IIV3/4 (Table 2).

**Table 2:** Vaccine effectiveness against influenza A of cell culture and egg-based inactivated influenza vaccines among 4-64-year-olds, Kaiser Permanente Northern California 2017–18.

		*Vaccine Effectiveness (VE) Against Influenza A*		
Comparison	Hazard Ratio^a^ (95% CI)	Relative VE (%) (95% CI)	Absolute VE (%) (95% CI)	P-value
ccIIV4 vs SD-IIV3/4 vaccinees	0.92 (0.77, 1.10)	8.0 (-10, 23)	n/a	0.35
ccIIV4 vaccinees vs unvaccinated	0.68 (0.57, 0.81)	n/a	31.7 (18.7, 42.6)	<0.0001
SD-IIV3/4 vs unvaccinated	0.80 (0.75, 0.86)	n/a	20.1 (14.5, 25.4)	<0.0001

CI, confidence interval; n/a, not applicable; ccIIV4, quadrivalent cell-culture inactivated influenza vaccine; SD-IIV3/4, trivalent or quadrivalent inactivated influenza vaccines.

^a^Cox regression with a calendar timeline, stratified by birth year. Adjusted for sex, facility, race/ethnicity, years of membership, prior season flu vaccine, diabetes, asthma, COPD, CHD, #IP admits in prior year, #weeks with OP visits in prior year.

The rVE against influenza B comparing ccIIV4 with SD-IIV3 was 39.6% (95% CI 27.9, 49.3). Compared with the unvaccinated, the adjusted aVE against influenza B was 40.9% (95% CI 30, 50.1) for ccIIV4 and 9.7% (95% CI 3.5, 15.6) for SD-IIV3 vaccines (Table 3).

**Table 3:** Vaccine effectiveness against influenza B of cell culture and egg-based inactivated influenza vaccines among 4-64-year-olds, Kaiser Permanente Northern California 2017–18.

		*Vaccine Effectiveness (VE) Against Influenza B*		
Comparison	Hazard Ratio^a^ (95% CI^2^)	Relative VE (%) (95% CI)	Absolute VE (%) (95% CI)	P-value
ccIIV4 vs SD-IIV3 vaccinees	0.60 (0.51,0.72)	39.6 (27.9, 49.3)	n/a	<0.0001
ccIIV4 vaccinees vs unvaccinated	0.59 (0.50, 0.70)	n/a	40.9 (30.0, 50.1)	<0.0001
SD-IIV3 vaccinees vs unvaccinated	0.90 (0.84, 0.97)	n/a	9.7 (3.5, 15.6)	0.0028

CI, confidence interval; n/a, not applicable; ccIIV4, quadrivalent cell-culture inactivated influenza vaccine; SD-IIV3, trivalent inactivated influenza vaccines.

^a^Cox regression with a calendar timeline, stratified by birth year. Adjusted for sex, facility, race/ethnicity, years of membership, prior season flu vaccine, diabetes, asthma, COPD, CHD, #IP admits in prior year, #weeks with OP visits in prior year.

In analyses subset by age, the estimate of rVE against influenza A was more favorable for ccIIV4 among 4 -<18-year olds (rVE 17.8, 95% CI -6.2, 36.4) than among 18-64-year olds (rVE -5.8, 95% CI -36.1, 17.7), but these confidence intervals are wide and this difference in rVE estimates could be due to chance alone. Neither vaccine was very effective against influenza A in adults; both vaccines were more effective against A in children/adolescents than in adults (Table 4).

**Table 4:** Vaccine effectiveness of cell culture and egg-based inactivated influenza vaccines against influenzas A and B stratified by age, Kaiser Permanente Northern California 2017–18.

		*Vaccine Effectiveness (VE)*		
Comparison	Hazard Ratio^a^ (95% CI)	Relative VE (%) (95% CI)	Absolute VE (%) (95% CI)	P-value
*Influenza A*				
ccIIV4 vs SD-IIV3/4 vaccinees				
4 - <18 year olds^b^	0.82 (0.64, 1.06)	17.8 (-6.2, 36.4)	n/a	0.13
18–64 year olds^c^	1.06 (0.82, 1.36)	-5.8 (-36.1, 17.7)	n/a	0.66
ccIIV4 vaccinees vs unvaccinated				
4 - <18 year olds	0.53 (0.42, 0.67)	n/a	46.8 (32.6, 58.1)	<0.0001
18–64 year olds	1.01 (0.78, 1.30)	n/a	-0.7 (-29.8, 21.9)	0.96
SD-IIV3/4 vaccinees vs unvaccinated				
4 - <18 year olds	0.64 (0.57, 0.72)	n/a	36.0 (28.2, 43.0)	<0.0001
18–64 year olds	0.90 (0.83, 0.98)	n/a	9.8 (1.7, 17.2)	0.02
*Influenza B*				
ccIIV4 vs SD-IIV3 vaccinees				
4 - <18 year olds	0.58 (0.47, 0.72)	42.3 (28.4, 53.5)	n/a	<0.0001
18–64 year olds	0.79 (0.58, 1.07)	21.4 (-7.3, 42.4)	n/a	0.13
ccIIV4 vaccinees vs unvaccinated				
4 - <18 year olds	0.54 (0.44, 0.66)	n/a	45.8 (33.7, 55.8)	<0.0001
18–64 year olds	0.72 (0.53, 0.99)	n/a	27.8 (1.2, 47.2)	0.04
SD-IIV3 vaccinees vs unvaccinated				
4 - <18 year olds	0.93 (0.85, 1.03)	n/a	6.7 (-2.8, 15.3)	0.16
18–64 year olds	0.85 (0.78, 0.94)	n/a	14.6 (6.1, 22.3)	0.001

CI, confidence interval; n/a, not applicable; ccIIV4, quadrivalent cell-culture inactivated influenza vaccine; SD-IIV3/4, trivalent or quadrivalent inactivated influenza vaccines.

^a^Cox regression with a calendar timeline, stratified by birth year and prior season flu vaccine.

^b^4-<18-year-old models also adjusted for sex, facility, race/ethnicity, years of membership, asthma, and number of weeks with OP visits in prior year.

^c^18-64 year old models also adjusted for sex, facility, race/ethnicity, years of membership, asthma, COPD, CHD, diabetes, number of weeks with OP visits in prior year, and #IP admits in prior year.

Supplemental analyses using the test-negative design yielded similar results, with an rVE against influenza A of 9.8% (95% CI -10.4, 26.3). The aVE was 39.9% (95% CI 26.6, 50.9) for ccIIV4 and 32.1% (95% CI 26.3, 37.4) for SD-IIV3/4 vaccines. The rVE of ccIIV4 vs SD-IIV3 against influenza B was 42.2% (95% CI 29.4, 52.7). The aVE was 50.0% (95% CI 39.3, 58.9) for ccIIV4 and 22.4% (95% CI 15.9, 28.5) for SD-IIV3 vaccines.

## Discussion

This study investigated whether the VE of ccIIV4 against influenza A differed from SD-IIV3/4 vaccines. Among 4-64-year olds during the 2017–18 influenza season, the relative vaccine effectiveness point estimate of ccIIV4 against influenza A was 8%. While the rVE point estimate hinted that ccIIV4 may be modestly more effective, the difference was not significant. Similarly, when compared with unvaccinated individuals, the absolute VE of ccIIV4 (31.7%) was a little higher than the absolute VE after SD-IIV3/4 (20.9%), but again the difference between the 2 vaccines was not significant. During the 2017–2018 influenza season, in which approximately 84% of the circulating influenza A in California was H3N2 (17, 18), neither vaccine was very effective; ccIIV4 was not much more effective than SD-IIV3/4 during this influenza season in which A(H3N2) predominated.

It has been hypothesized that the lower VE estimates against A(H3N2) in recent years may have been related to genetic changes which could have occurred on the hemagglutinin protein component of the influenza A(H3N2) vaccine virus as it was passaged in eggs [10, 23, 24]. Such genetic changes to the vaccine viral hemagglutinin protein could potentially result in an immune response to egg-based vaccination that would be less effective against circulating influenza A(H3N2) viruses. Animal model studies have indicated that cell culture-derived antigens provide better immune response which could lead to a better vaccine effectiveness [25, 26]. However, a recent study reported that following ccIIV4, antibody titers were low to all H3N2 viruses during the 2017–18 season [27]. Our study did not find evidence of a large difference between ccIIV4 and SD-IIV3/4 vaccines, suggesting that the low VE against influenza A(H3N2) during 2017–18 may not be fully explained by the passaging of vaccine viruses in eggs [28].

Our study found that during the 2017–18 influenza season, ccIIV4 was 40% more effective against influenza B than were SD-IIV3 vaccines. When compared with unvaccinated, both ccIIV4 and SD-IIV3 vaccines were effective against influenza B (nearly 41% for ccIIV4 and 10% for SD-IIV3). The relative and absolute VEs against influenza B were also higher after ccIIV4 than after and SD-IIV3, which would be expected since ccIIV4 contained both Yamagata and Victoria B lineages, versus SD-IIV3 which only contained B/Victoria. In California during 2017–18 influenza season, only 13% of tested circulating influenza B was Victoria lineage [17]. These results both lend credibility to our overall rVE analyses and demonstrate that inclusion of the B/Yamagata in the quadrivalent vaccine provided substantially better protection against influenza B in a year with predominant Yamagata lineage virus.

In recent years, the VE of egg-based standard dose vaccines against influenza A(H3N2) have trended higher among younger individuals than in older people [1, 29, 30]. The reasons for this have not been clear, though one idea is that it may be related to children having lower pre-vaccination antibody titers to A(H3N2) and therefore increased immune responses after vaccination [5]. Consistent with prior studies, we also observed that the aVE against influenza A was significantly higher for those aged 4 to 18 years than for those aged 18–65 years for both ccIIV4 (46.8% vs -0.7%) and SD-IIV3/4 vaccines (36% vs 9.8%). We also found that the rVE of ccIIV4 versus SD-IIV3/4 vaccines against influenza A trended higher for those aged 4 to 18 years (17.8%) than for those 18+ years (-5.8%), though the difference was not significant. This was somewhat surprising because if, according to the theory that the SD-IIV3/4 vaccines may be less effective in adults, one might expect that the relative benefit of ccIIV4 might be greater in adults. In contrast, while not definitive as this study was not powered to detect small differences, our results suggested higher relative benefit for ccIIV4 in younger people.

Our findings are consistent with a larger recent study which estimated the rVE of ccIIV4 versus SD-IIV3/4 vaccines of 10% (95% CI 7%, 13%) in a Medicare population aged >65 years [31]. While both studies had a low proportion who received ccIIV4 (5% in their study vs 8.3% in ours), their larger population yielded a more precise estimate of rVE. Our study included a different age range of 4–64 year olds, which allowed us to age-stratify the analyses and investigate for differences between children/adolescents and adults. Another important difference was the influenza case definition used. Our study defined a case as having a PCR positive influenza test, while the Medicare study was limited to influenza-related hospital encounters identified using only diagnostic codes which could have been subject to misclassification bias. Finally, because the KPNC influenza PCR test always assesses for both influenza A and B, we also estimated both absolute and relative VE for ccIIV4 and SD-IIV3/4 vaccines against both influenzas A and B. A recent study conducted by the Department of Defense suggested that, although their results were not statistically significant, ccIIV4 may have offered better protection against influenza A(H3N2) during 2017–18 [32]. In contrast, another study did not find that ccIIV4 offered significantly better protection against than did SD-IIV3/4 against influenza hospitalizations [33].

A strength of our relative VE analyses was that both analyses were restricted to vaccinees to avoid confounding related to differences between unvaccinated and vaccinated persons. Since all vaccinees had utilized care in recent months, our rVE analyses limited the differences between ccIIV4 and SD-IIV3/4 vaccinees in their propensity to be tested when sick. It is reassuring that results of the cohort and the test-negative analyses were consistent because it indicates that our cohort analysis was able to carefully adjust for healthcare seeking behaviors. We also carefully adjusted for age. By using a calendar timeline in our Cox regression analysis, we carefully controlled for calendar time by day, which was essential because the proportion of persons vaccinated with either ccIIV4 or SD-IIV3/4 changed over time during the influenza season.

This study had limitations. Although KPNC population is large, KPNC had relatively limited use of ccIIV4 during 2017–18 season. Power was limited because only 84,420 individuals in the study population (8.3% of vaccinees) received ccIIV4. The 95% confidence interval surrounding our estimate of rVE against influenza A extended from -10% to 23%. Thus, we cannot rule out the null hypothesis (i.e., that rVE = 0), although we also cannot rule out the possibility that rVE is in the 10 to 20% range which could have substantial public health benefit. In addition, the effectiveness of influenza vaccines can vary in different populations and different influenza seasons. There was also potential for bias in the rVE analyses if providers preferentially gave either vaccine to higher risk individuals, although this was unlikely to occur systematically because neither KPNC nor the CDC expressed preferential recommendations for either vaccine, and in the aVE analyses if propensity to seek care differed between vaccinated and unvaccinated. We also did not adjust the analyses for the setting of sample collection. However, we think it is unlikely that this was an important confounder in the rVE analyses because setting would need to be associated with the type of vaccine received rather than with vaccination itself. The supplemental test negative analyses were also susceptible to bias if there was variation in illness severity between those with PCR-positive influenza and those with PCR-negative non-influenza disease or if vaccine status was misclassified due to some individuals receiving an influenza vaccine outside of KPNC. Further, we were only able to adjust for calendar month in the test negative analyses due to sparser data. Finally, we did not have data on the influenza subtype strains. Although KPNC comprises a substantial portion of the state, we did rely on California state data to infer that the predominant strains in our study population were A(H3N2) and B/Yamagata.

In conclusion, this study found that the cell-culture influenza vaccine was not much more effective than standard dose egg-based influenza vaccines. Both ccIIV4 and SD-IIV3/4 vaccines were not very effective during the 2017–2018 influenza season in which A(H3N2) predominated. The ccIIV4 vaccine was more effective against influenza type B and inclusion of the B/Yamagata strain seemed to provide substantially better protection against influenza B in a year with predominant Yamagata lineage virus. This study highlights the need for ongoing monitoring of vaccine effectiveness and ultimately better influenza vaccines.

## Supporting information

S1 Fig Influenza Vaccine Coverage in Study Population by Week, Kaiser Permanente Northern California 2017–18. Graph on the left represents standard dose egg-based inactivated influenza vaccine (SD-IIV) coverage. SD-IIV includes both trivalent and quadrivalent vaccines. Graph on the right represents quadrivalent cell-culture inactivated influenza vaccine coverage.
(EPS)

S2 Fig Percent of all Influenza Vaccines that was Cell-Culture Inactivated Influenza Vaccine (ccIIV4) by Week, Kaiser Permanente Northern California 2017–18. (EPS)
